# A comparative study of uniportal video-assisted thoracic surgery versus thoracotomy in the treatment of TB empyema

**DOI:** 10.5588/ijtldopen.26.0017

**Published:** 2026-08-11

**Authors:** H. Liu, P. Xu, S. Zheng, X. Wang, Y. Long, S. Lin, Y. Wang, J. Zeng, M. Fang, K. Qiao, S. Lu, H. Wang

**Affiliations:** The Third People’s Hospital of Shenzhen, Department of Thoracic Surgery, National Clinical Research Center for Infectious Disease, The Second Affiliated Hospital of Southern University of Science and Technology, Shenzhen, Guangdong, China.

**Keywords:** tuberculosis, pleural decortication, VATS, surgical outcomes

## Abstract

**BACKGROUND:**

Chronic TB empyema is a serious complication of TB that often requires surgery because drug penetration into fibrotic pleural tissue is poor. We compared uniportal video-assisted thoracic surgery (VATS) and thoracotomy for pleural decortication.

**METHODS:**

We retrospectively analysed 112 patients with chronic TB empyema who underwent surgical treatment between 2020 and 2024. Thoracic morphometric changes were assessed using pre- and postoperative computed tomography, and factors associated with postoperative thoracic expansion were explored.

**RESULTS:**

Of the 112 patients, 65 underwent thoracotomy and 47 uniportal VATS. The overall excellent/good outcome rate was 91.9%, based on postoperative lung re-expansion, residual empyema cavity and residual fibrous pleural plaque. Compared with thoracotomy, uniportal VATS was associated with less intraoperative blood loss (151.8 ± 127 vs. 278 ± 231 mL, *P* < 0.001) and a shorter postoperative hospital stay (8.2 ± 3.7 vs. 9.9 ± 3.1 days, *P* = 0.002). Both groups showed significant postoperative improvement in thoracic morphometric measurements. Older age was associated with poorer postoperative thoracic expansion.

**CONCLUSION:**

In this retrospective single-centre cohort, uniportal VATS was associated with more favourable perioperative outcomes than thoracotomy, with comparable short-term radiographic recovery.

TB empyema is a severe complication of *Mycobacterium tuberculosis* infection, usually arising as a consequence of pulmonary TB. It is characterised by the accumulation of purulent material within the pleural space, accompanied by chronic inflammation and progressive fibrotic change of the pleura. This process can markedly impair respiratory function and, in advanced cases, may lead to chest wall invasion, rib destruction, and extension of infection to adjacent or distant sites.^1,2^ Anti-TB chemotherapy remains the cornerstone of treatment for TB empyema.^3^ However, in the fibrotic stage, the thickened pleural peel and fibrocalcific wall may limit drug penetration and reduce treatment efficacy.^3,4^ According to thoracic surgery guidelines, surgical decortication should be considered when pleural drainage and medical treatment fail to achieve adequate control.^5,6^ Open thoracotomy is effective, but is associated with greater surgical trauma and slower postoperative recovery. Uniportal video-assisted thoracic surgery (VATS) offers a less invasive alternative and may improve perioperative recovery; however, comparative evidence in TB empyema remains limited.^7–11^

We retrospectively compared uniportal VATS and thoracotomy for pleural decortication in patients with chronic TB empyema treated at our centre over a 4-year period. We aimed to evaluate perioperative and short-term radiographic outcomes and to explore factors associated with postoperative thoracic expansion.

## METHODS

This retrospective study was conducted at Shenzhen Third People’s Hospital, a tertiary TB-designated hospital in Shenzhen, China. Medical records of patients with TB empyema treated between June 2020 and June 2024 were reviewed. During 2020–2021, thoracotomy was the routine surgical approach at our institution; after 2022, uniportal VATS became the predominant approach as the minimally invasive decortication programme matured. Thus, treatment allocation mainly reflected changes in institutional practice over time. All operations were performed by the same thoracic surgery team. Inclusion criteria were: 1) confirmed or clinical diagnosis of chronic TB empyema based on history, symptoms, and characteristic computed tomography (CT) findings; 2) persistent pleural cavity or symptoms despite ≥2 months of standard anti-TB chemotherapy with drainage; 3) controlled systemic infection; and 4) preoperative assessment suggesting potential lung re-expansion after decortication. Exclusion criteria were: 1) excessive anaesthetic or operative risk; 2) uncontrolled systemic infection; 3) adequate response to ongoing medical treatment; 4) irreversible end-stage lung destruction or inability of the affected lung to re-expand; and 5) drug-resistant TB. Drug-resistant cases were excluded to minimise the potential impact of poor or heterogeneous anti-TB treatment response on postoperative recovery.

Before surgery, all included patients received standard four-drug anti-TB therapy for >2 months: isoniazid, rifampicin, ethambutol, and pyrazinamide. After surgery, rifampicin, isoniazid, and ethambutol were continued for 9–12 months.

### Definitions of morphological changes

To quantify thoracic morphometric changes after pleural decortication, non-contrast chest CT scans obtained within 1 week before surgery and 6 months after surgery were compared. The axial plane at the level of the largest empyema cavity was used as the standard measurement plane, as it represented the site of maximal pleural space occupation and lung compression and allowed consistent within-patient comparison.

**Figure 1.**
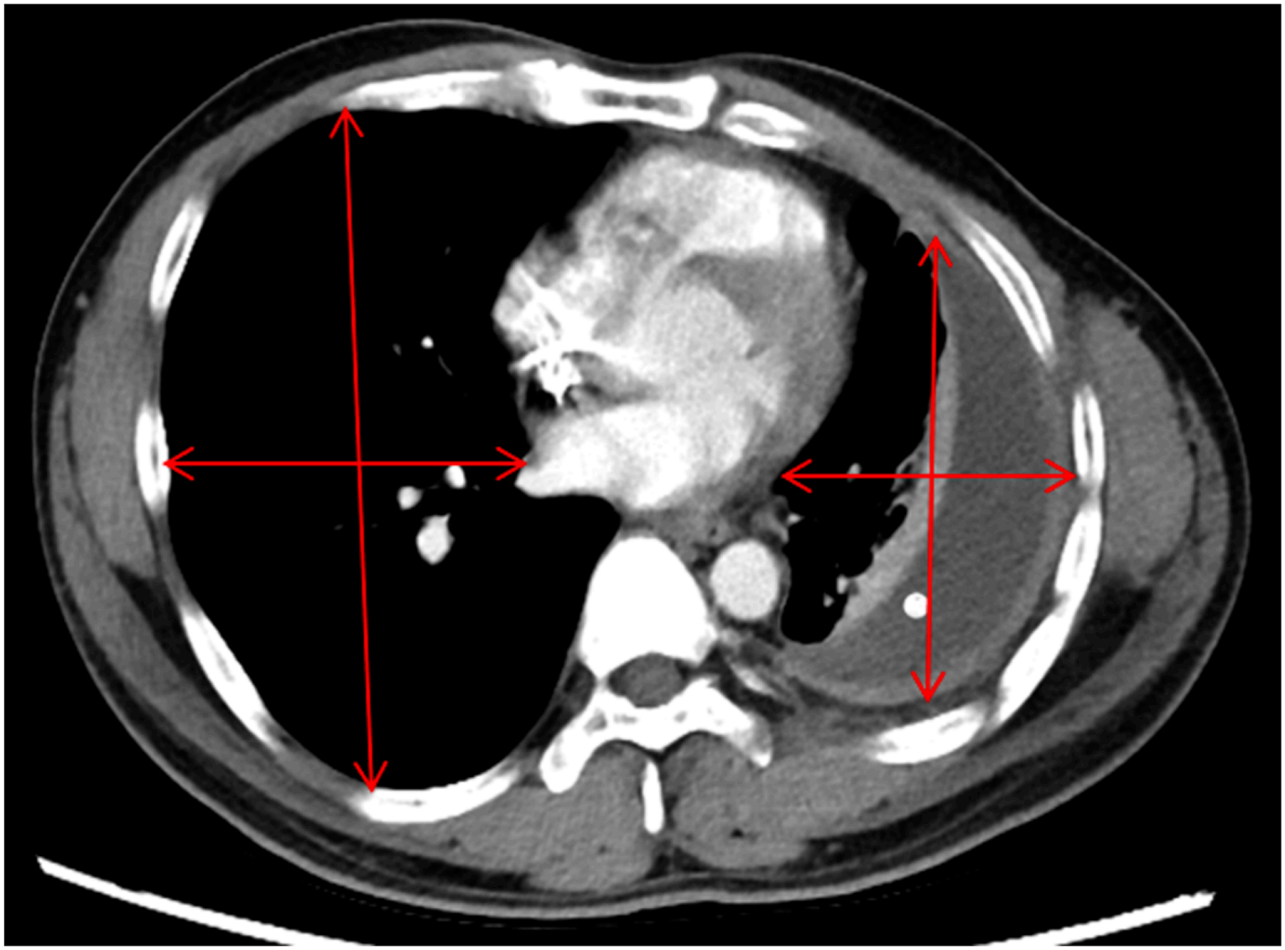
Quantitative assessment of thoracic morphological changes in patients with chronic TB empyema after pleural decortication using non-contrast chest computed tomography (CT). Preoperative CT shows right hemithoracic collapse, pleural thickening and calcification, right-sided loculated pleural effusion, and marked compression with poor expansion of the affected lung. Affected-side transverse diameter was measured from the inner edge of the most medial rib on the affected chest wall to the junction of the mediastinal pleura and the lung on the affected side. Unaffected-side transverse diameter was measured from the corresponding junction on the unaffected side to the inner edge of the most medial rib on the unaffected chest wall. Affected-side anteroposterior diameter was measured from the inner edge of the most anterior rib to the inner edge of the most posterior rib on the affected chest wall. Unaffected-side anteroposterior diameter was measured from the inner edge of the most anterior rib to the inner edge of the most posterior rib on the unaffected chest wall. The horizontal line indicates the transverse diameter, and the vertical line indicates the anteroposterior diameter.

Four parameters were measured on the selected axial image (Figure 1): affected-side transverse diameter (ATD), unaffected-side transverse diameter (UTD), affected-side anteroposterior diameter (AAPD), and unaffected-side anteroposterior diameter (UAPD). ATD was defined as the distance from the inner edge of the most medial rib of the affected hemithorax to the junction of the mediastinal pleura and lung on the affected side, and UTD as the corresponding distance on the unaffected side. AAPD was defined as the distance from the inner edge of the most anterior rib to the inner edge of the most posterior rib on the affected side, and UAPD as the corresponding distance on the unaffected side. The same method was used for postoperative CT assessment.^12^ Morphometric change was expressed as the preoperative-to-postoperative differences in ATD, UTD, AAPD, and UAPD, as well as the total change across all four measurements.

### Outcome assessment

Postoperative outcomes were graded as excellent, good, fair, or poor according to lung re-expansion, residual empyema cavity, and residual fibrous pleural plaque on postoperative imaging. Excellent was defined as complete lung re-expansion with no residual empyema cavity or fibrous pleural plaque. Good was defined as complete lung re-expansion with no residual empyema cavity and minimal residual fibrous pleural plaque. Fair was defined as >50% lung re-expansion, >50% reduction in empyema cavity, and <50% residual fibrous pleural plaque. Poor was defined as <50% lung re-expansion, <50% reduction in empyema cavity, and >50% residual fibrous pleural plaque. This grading framework was informed by a previously published study of surgical treatment for TB empyema.^10^ Postoperative outcome grading was independently performed by two senior thoracic surgeons based on imaging findings and follow-up records. Disagreements were resolved by consensus.

### Statistical analysis

Categorical variables were presented as n (%), and continuous variables as mean ± standard deviation (SD). Between-group comparisons were performed using the χ^2^ test or Fisher’s exact test for categorical variables, the Student’s *t* test for normally distributed continuous variables, and the Wilcoxon rank-sum test for non-normally distributed continuous variables. For exploratory analysis, patients were stratified by total Δ (<2 cm vs. ≥2 cm). The 2-cm threshold was selected pragmatically on the basis of the distribution of thoracic dimensional change in this cohort and was used for exploratory stratification rather than as a prespecified or validated clinical cut-off. All analyses were performed using Stata version 14.0, and a two-sided *P* value <0.05 was considered statistically significant.

### Ethical statement

The study was approved by the Ethics Committee of Shenzhen Third People’s Hospital (No. 2025-198-01) and conducted in accordance with the Declaration of Helsinki. The requirement for informed consent was waived because of the retrospective design.

## RESULT

Between June 2020 and June 2024, 112 patients with TB empyema underwent surgery at Shenzhen Third People’s Hospital. The cohort comprised 87 men and 25 women, with a mean age of 36.4 ± 15.8 years (range 5–85). Of these, 65 underwent thoracotomy with pleural decortication and 47 underwent uniportal VATS (Table 1).

**Table 1.** Comparative analysis of thoracotomy and uniportal video-assisted thoracoscopic surgery (VATS) in the treatment of TB empyema patients.

Characteristics	Thoracotomy surgery	Uniportal VATS	Total	*P* value
	n = 65	n = 47	n = 112	
Age (years)	37.2 ± 15.0	35.2 ± 16.9	36.4 ± 15.8	0.267
Gender				
Men	51 (78.5%)	36 (76.6%)	87 (77.7%)	0.815
Women	14 (21.5%)	11 (23.4%)	25 (22.3%)	
BMI	21.0 ± 3.4	22.1 ± 3.3	21.4 ± 3.4	0.019
Surgical site				
Left	24 (36.9%)	23 (48.9%)	47 (42.0%)	0.475
Right	38 (58.5%)	23 (48.9%)	61 (54.5%)	
Bilateral	3 (4.6%)	1 (2.2%)	4 (3.5%)	
Transverse plane				
ΔATD (cm)	1.14 ± 065	0.81 ± 0.82	1.00 ± 0.74	0.020
ΔAAPD (cm)	1.51 ± 1.08	1.23 ± 0.81	1.39 ± 0.98	0.135
ΔUTD (cm)	0.10 ± 0.40	0.17 ± 0.55	0.13 ± 0.46	0.469
ΔUAPD (cm)	0.29 ± 0.53	0.49 ± 0.61	0.38 ± 0.57	0.072
Total Δ (cm)	3.04 ± 1.88	2.70 ± 1.79	2.90 ± 1.85	0.327
Duration of preoperative symptoms (months)	10.9 ± 15	17.3 ± 41	13.6 ± 29	0.234
Surgery duration (minutes)	223.9 ± 54	216.1 ± 100	220.6 ± 77	0.153
Intraoperative blood loss (mL)	278 ± 231	151.8 ± 127	225 ± 203	<0.001
Postoperative hospital stays (days)	9.9 ± 3.1	8.2 ± 3.7	9.2 ± 3.5	0.002
Postoperative drainage volume (mL)	878.2 ± 514	885.7 ± 1,091	881.4 ± 804	0.140

BMI = body mass index; ATD = affected-side transverse diameter; UTD = unaffected-side transverse diameter; AAPD = affected-side anteroposterior diameter; UAPD = unaffected-side anteroposterior diameter.

### Comparison of perioperative outcomes between uniportal VATS and thoracotomy

**Figure 2.**
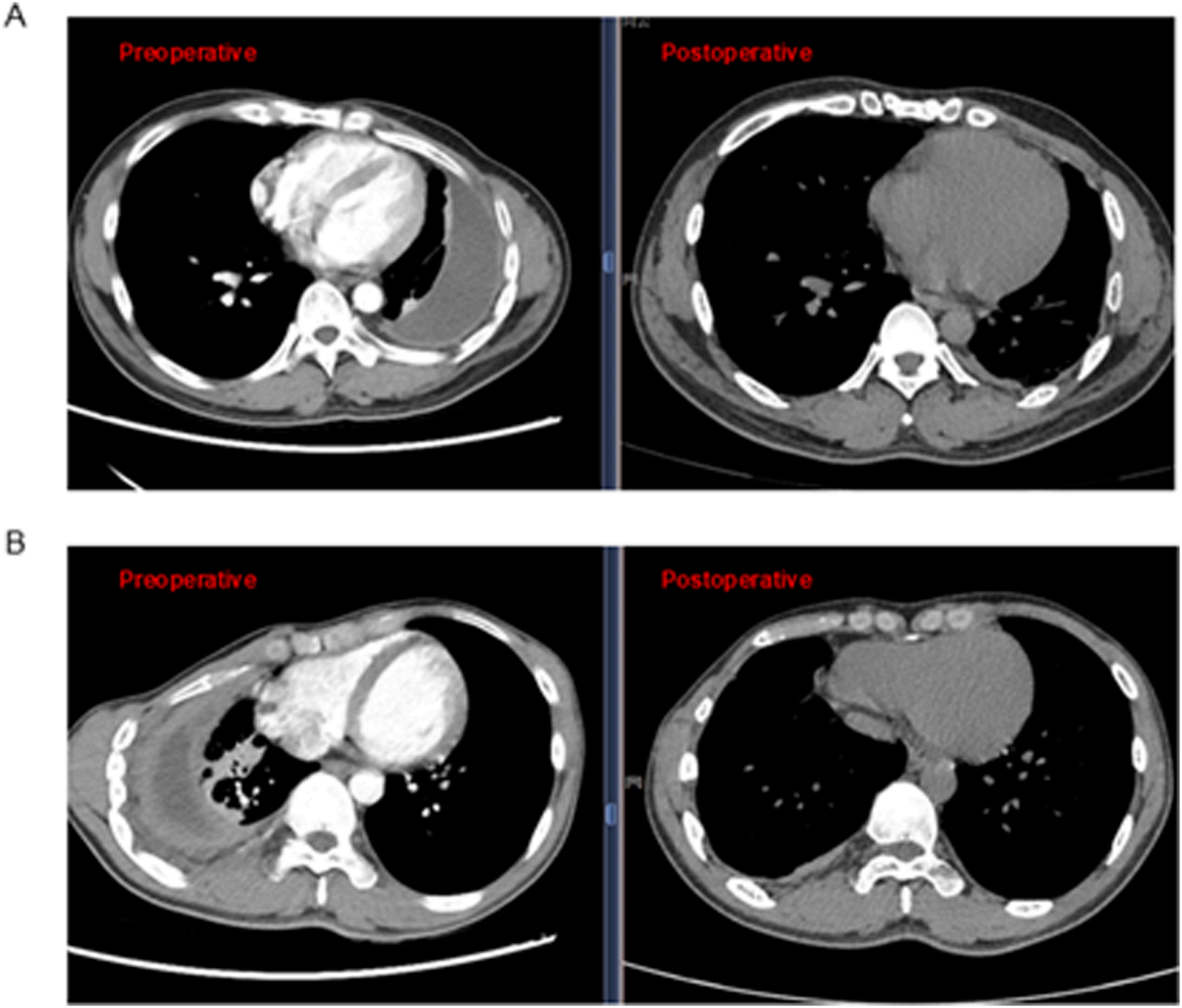
Representative chest radiographs before and after pleural decortication in patients treated with thoracotomy and uniportal video-assisted thoracoscopic surgery (VATS). **A:** Preoperative and postoperative radiographs from a representative patient undergoing thoracotomy. **B:** Preoperative and postoperative radiographs from a representative patient undergoing uniportal VATS.

Perioperative variables were compared between the thoracotomy and uniportal VATS groups. Uniportal VATS was associated with significantly less intraoperative blood loss than thoracotomy. Postoperative hospital stay was also significantly shorter in the uniportal VATS group. No significant between-group differences were observed in operative time, postoperative drainage volume, or short-term radiographic recovery (Table 1). Representative preoperative and postoperative chest radiographs from the two surgical approaches are shown in Figure 2.

### CT-based morphometric changes after surgery

To evaluate postoperative thoracic remodelling and radiographic lung re-expansion, we compared the transverse and anteroposterior diameters of the affected and unaffected hemithoraces on CT before surgery and 6 months after surgery. On the affected side, the transverse diameter increased from 9.5 ± 1.0 cm preoperatively to 10.5 ± 1.1 cm postoperatively (*P* < 0.001), and the anteroposterior diameter increased from 12.8 ± 1.9 cm to 14.2 ± 1.9 cm (*P* < 0.001). On the unaffected side, the transverse diameter changed from 11.3 ± 1.3 cm to 11.4 ± 1.3 cm (*P* = 0.005), and the anteroposterior diameter from 15.1 ± 1.8 cm to 15.5 ± 1.8 cm (*P* < 0.001). All four measurements showed significant postoperative changes, indicating improved thoracic morphometric expansion after surgery (Table 2).

**Table 2.** Radiographic morphometric changes before and after surgery in patients with TB empyema.

Transverse plane	Preoperative	Postoperative	*P* value
ATD (cm)	9.5 ± 1.0	10.5 ± 1.1	<0.001
AAPD (cm)	12.8 ± 1.9	14.2 ± 1.9	<0.001
UTD (cm)	11.3 ± 1.3	11.4 ± 1.3	0.005
UAPD (cm)	15.1 ± 1.8	15.5 ± 1.8	<0.001

ATD = affected-side transverse diameter; UTD = unaffected-side transverse diameter; AAPD = affected-side anteroposterior diameter; UAPD = unaffected-side anteroposterior diameter.

### Clinical follow-up outcomes and postoperative complications

All 112 patients completed 12 months of postoperative follow-up. Overall, 58 patients had an excellent outcome, 45 had a good outcome, and 9 had a fair outcome, yielding an excellent/good rate of 91.9%. Postoperative complications occurred in 19 patients (17.0%), including prolonged air leak lasting >2 weeks in six patients, loculated pleural effusion requiring percutaneous drainage in six, and delayed wound healing due to incision infection in seven. In patients with more than one complication, only the complication with the greatest impact on prognosis was recorded. No patient required reoperation for recurrence, and no mortality was observed during follow-up.

### Factors associated with suboptimal thoracic expansion after decortication

To explore factors associated with postoperative thoracic expansion, the 112 patients were stratified into two groups according to the total postoperative change in the four thoracic diameters, using an exploratory cut-off of 2 cm. Thirty-seven patients had a total Δ < 2 cm and 75 had a total Δ ≥ 2 cm. The two groups were compared with respect to age, body mass index, duration of preoperative symptoms, operative time, intraoperative blood loss, postoperative drainage volume, and postoperative hospital stay. In this exploratory analysis, older age was associated with less favourable postoperative thoracic expansion (Table 3).

**Table 3.** Factors associated with postoperative thoracic dimensional change (total Δ).

Characteristics	Total Δ < 2 cm	Total Δ ≥ 2 cm	*P* value
	n = 37	n = 75	
Age (years)	42.6 ± 14.8	33.3 ± 15.4	0.001
Gender			
Men	30 (81.1%)	57 (76.0%)	0.544
Women	7 (18.9%)	18 (24.0%)	
BMI	22.0 ± 3.6	21.2 ± 3.3	0.197
Surgical site			0.134
Left	11 (29.7%)	36 (48.0%)	
Right	24 (64.9%)	37 (49.3%)	
Bilateral	2 (5.4%)	2 (2.7%)	
Surgical method			0.549
Thoracotomy surgery	20 (54.1%)	45 (60.0%)	
Uniportal VATS	17 (45.9%)	30 (40.0%)	
Duration of preoperative symptoms (months)	18.9 ± 44.0	11.0 ± 17.2	0.418
Surgery duration (minutes)	241.6 ± 102.3	210.3 ± 58.6	0.126
Intraoperative blood loss (mL)	230.9 ± 254.2	222.1 ± 183.2	0.526
Postoperative hospital stays (days)	8.9 ± 4.4	9.3 ± 3.0	0.177
Postoperative drainage volume (mL)	1,086.9 ± 1,230.3	780.0 ± 448.5	0.492

BMI = body mass index; VATS = video-assisted thoracoscopic surgery.

## DISCUSSION

In this retrospective single-centre cohort, uniportal VATS was associated with less intraoperative blood loss and a shorter postoperative hospital stay than thoracotomy, while short-term radiographic recovery was comparable between the two groups. In addition, exploratory analysis suggested that older age was associated with less favourable postoperative thoracic expansion. These findings indicate that uniportal VATS may offer perioperative advantages in selected patients with chronic TB empyema, although the study design does not allow conclusions regarding superiority.

Pulmonary function assessment is a more direct measure of postoperative recovery. However, because postoperative pulmonary function follow-up data were incomplete in this retrospective cohort, CT-based thoracic morphometric measurements were used as supportive radiographic indicators of postoperative lung re-expansion and thoracic remodelling. Previous studies have suggested that CT-based morphologic parameters may help assess postoperative structural recovery and predict clinically relevant outcomes after decortication for TB empyema.^10,13,14^ In this context, CT measurements provided a practical reference for postoperative evaluation in the present study. Nevertheless, these imaging findings should be interpreted as supplementary rather than definitive clinical endpoints and should be considered together with clinical follow-up outcomes and postoperative complications.

The perioperative findings of the present study are consistent with the expected advantages of a minimally invasive approach. Compared with thoracotomy, uniportal VATS was associated with reduced intraoperative blood loss and a shorter postoperative hospital stay, suggesting less operative trauma and faster early recovery. Similar perioperative advantages of thoracoscopic approaches have been reported in previous studies of TB empyema.^9,10,15,16^ At the same time, no significant difference was observed in short-term radiographic recovery between the two approaches. These results suggest that uniportal VATS may be a feasible minimally invasive option for pleural decortication in selected patients with chronic TB empyema.^12,16^ However, given the retrospective design and limited control for confounding, these findings should be interpreted as observational associations rather than evidence that uniportal VATS is superior to thoracotomy.

This study has several important limitations. First, it was a retrospective single-centre study, and the choice of surgical approach largely reflected a temporal transition in institutional practice, with thoracotomy used mainly in the earlier period and uniportal VATS in the later period. Therefore, residual confounding related to time trends, perioperative care, and increasing surgical experience cannot be excluded. Second, CT-based thoracic measurements were used as supportive radiographic indicators, whereas pulmonary function data, a more direct measure of postoperative recovery, were incomplete in this cohort. In addition, the 2-cm threshold used for total Δ stratification was selected pragmatically from the observed distribution in this dataset and was intended for exploratory analysis rather than as a prespecified or validated clinical cut-off. Therefore, the related findings should be interpreted cautiously. Third, follow-up was limited to 12 months, and longer-term recurrence, residual thoracic deformity, and functional outcomes were not fully assessed.

## CONCLUSIONS

Uniportal VATS was associated with favourable perioperative outcomes and comparable short-term radiographic recovery in this cohort. However, these findings should be interpreted cautiously and require confirmation in prospective studies with longer follow-up and standardised functional evaluation.
